# Treatment of Children's Teeth

**Published:** 1884-03

**Authors:** E. T. Darby

**Affiliations:** Philadelphia, Pa.


ARTICLE IV.

### TREATMENT OF CHILDREN'S TEETH.

BY PROF. E. T. DARBY, PHILADELPHIA, PA.

(Read before the New York Odontological Society.)

*Mr. President and Gentlemen* :—It was with great reluctance that I consented to appear before you this evening. The idea of my presuming to instruct such a body of gentlemen as I see before me is absurd, to say the least. Will you not therefore accept as my apology for the apparent audacity, my desire to gratify your worthy president, as well as the importance of the subject chosen ?

That the teeth of children are not receiving that degree of attention which they demand is a fact I feel sure none will attempt to deny. I am confident I speak with moderation when I say one-half of the children of the present day are receiving little, if any, dental treatment prior to their tenth year. There seems to be a belief quite prevalent that the temporary teeth are of little importance, and that since their period of duration is a brief one they are unworthy of the attention which is bestowed upon the permanent set. That the dental profession is largely to blame for this wide-spread belief I am free to assert. Each profession is in a measure responsible for the ignorance which exists in the public mind with reference to itself. No stream is higher than its fountain-head, neither is a community wiser in the arts and sciences than it is made by those who are the recognized exponents of them. Believing the subject worthy of our attention. I ask your indulgence for a few moments only.

And first the argument.

The temporary teeth are of vital importance to the well-being of the child, and ought therefore to be preserved. They are important physiologically. Their eruption is co-incident with the child's need of them. The period of milk-diet has passed, and food of a solid nature enters more or less largely into the daily needs of the growing child. The act of mastication is one of great importance in the digestive function, and yet, how few children are able to properly masticate their food. Take the average child between the third and seventh year and note with what difficulty it masticates solid food. It is constantly embarrassed in its efforts to do so because of the sensitiveness incident to decay in the molars. The food is carried from one side of the mouth to the other, and generally swallowed before it is half masticated. I doubt not one-half of the gastric troubles so common in children could be traced to imperfect mastication of the food taken in the stomach. The cause of this is due largely to imperfect teeth.

Again, the preservation of the temporary teeth is important from a humane standpoint. Consider for a moment the pain and suffering which children are daily and hourly experiencing as the result of carious teeth. The cry of pain comes alike from palace and hovel, and, I regret to say, too often meets with little or no sympathy on the part of those who are responsible for its cause. I believe it wholly unnecessary that any person living in this enlightened day should suffer an hour's pain from toothache. Our children should go from cradle to grave, though forty years intervene, without an exposed or aching pulp.

Again, the preservation of the temporary teeth is important from an æsthetic standpoint. What is more beautiful than twenty white teeth in the mouth of a child, and what more unsightly than a mouthful of diseased or discolored ones? I am aware that all teeth are not developed alike beautiful, but there is less deformity in the temporary than in the permanent set. A little care on the part of the parent and dentist in the early life of the child will insure white and beautiful teeth, and its appearance be rendered attractive rather than repulsive Children are not taught early enough in life the habit of cleansing their teeth. If children can be taught to read at the third or fourth year, they certainly can be taught to use the tooth-brush. Children are by very nature imitative. We all know how easily habits are formed in early life. An act is sown, a habit is reaped; a habit is sown, a character is reaped; a character is sown, a destiny is reaped.

Having considered briefly the importance of saving the teeth of children, let us next consider some of the difficulties.

I have already alluded to one of the chief difficulties which beset us, namely, ignorance on the part of the public. Parents tell us they did not know their children's teeth needed attention. It is their business to know it. There is an ignorance that is criminal. I hold that it is criminal for a mother not to know that scarlet fever and smallpox are contagious. It is criminal not to know that arsenic and corrosive sublimate and Paris green are poisonous. Ignorance and stupidity have been the cause of as much suffering and misery as intentional sin. Other parents will tell you they did not know their children's teeth needed attention, and they had intended having them cared for. Good intentions alone will not save teeth. There is a place, we are told, of which Diabolus is king, and where he holds glittering court, the streets of which are paved with good intentions. Every practitioner of dentistry should be a teacher as well, and his whole duty has not been preformed until each mother whom he can influence is made to realize the importance of early attention to her child's teeth.

Another great difficulty which we encounter in our dealings with children is the deception which has been practiced upon them at home. I sometimes think the nursery is a training-school for deception and falsehood. It is astonishing to what an extent deception is practiced upon children. For instance, they are told that the apothecary's physic is as sweet as sugar, that the surgeon's knife is as the scratch of a pin, and that the dentist's forceps are as painless as the barber's shears. Children are innately trustful and confiding, but when they have once been deceived it is difficult to gain their confidence. Parents will go home from a sitting at the dentist's and, in the presence of their children, complain of the instruments of "torture" and the pain they have experienced, producing in the mind of the child an intense fear of the dentist; and yet, the following day drag the same child to the same dentist for the performance of a similar operation, and assert, with an air of the greatest truthfulness, that it will not hurt them in the least. Truth is always better than falsehood. The confidence of a child is often worth more than that of an adult.

Children will bear a great deal of pain, and bear it patiently, if they are not deceived at the outset: but woe to him who, having first deceived his little patient, tries to re-establish its faith in him.

Permit me to relate a case which illustrates the truth of this. Some twelve or fourteen years ago a mother called with her daughter, a child of twelve years, to ask me if I would undertake the care of her teeth. She had already been to other dentists, but they failed to accomplish anything for her because of her intense fear and the nervous dread which the dentist produced upon her. I learned from the mother that years before, when it became necessary to remove a deciduous tooth, she had been taken to a dentist, and, without telling the child what he was about to do, he concealed his forceps in his sleeve and without her consent removed the tooth. She had been assured by him that she would not be hurt. To convince that child that all dentists are not liars was as difficult as it was tedious. All that was accomplished at the first and second sittings was the removal of a little stain from the teeth with a piece of wood and pumice. At the fifth or sixth sitting a temporary filling was allowed, and at subsequent sittings all of the teeth that needed filling were attended to. I had gained her confidence, and for at least twelve or fourteen years she has been a regular patient, and one of the most appreciative in my practice.

I know of nothing more diabolical than deceiving an innocent, trustful child, and he who will intentionally do it is an ignoble fellow. Another great difficulty is to be found in the dentist himself. He is not fond of children and dislikes to work for them; he is impatient and generally disagreeable. Children are quick to perceive this tendency, and at the hands of such a one will bear less pain than from another who impresses them differently. Such dentists do as little as possible for children, and pronounce them finished ere they have hardly begun. Again, children are often discouraged by too long sittings or too protracted operations. Half-hour sittings are long enough, and fifteen minutes often better. A great victory has been gained, if the child be sent away the first time with pleasant impressions. I remember to have once heard a dentist say he kept in his office a lot of china dolls which he gave to little girls at the close of the sitting for good behavior, also some cheap knives and rubber balls which he gave the boys. The principle is a good one, and if the dentist can thus ingratiate himself into the affections of the child, it is a good investment.

Having considered the importance of saving the teeth of children, likewise the difficulties, let us now attend to the methods.

I have already alluded to the importance of seeing the child early in life. If we would be of greatest benefit to children we must see them early in life and at frequent intervals. If the teeth are carious they should be filled while the cavities are yet small. A great mistake is often made by both parent and dentist by postponing such operations. Small cavities may be filled with little or no pain, and not infrequently superficial caries can be removed and the disease arrested. When the incisors are found slightly decayed, my practice has been to file them largely asunder, and afterwards polish the approximal surface with emery strips and oxide of tin. The same practice may be used to advantage on the approximal surfaces of the molars. If caries has penetrated the dentine and is too extensive to warrant removal, fill with gutta-percha, or what I consider superior, some of the better preparations of phosphate of zinc. And just here let me say, to get the best results with this material, it should be mixed very thick. I am in the habit of mixing it as thick as putty, and then rolling it between my thumb and finger, working the oxide of zinc into the mass until it will hold no more, and while in this condition pack it into the cavity as stiff as possible. But a few minutes is required for hardening, and the process may be hastened by throwing upon it a blast of hot air from the hot air syringe.

It frequently happens that the pulps are found exposed in the temporary molars. When such is the case I devitalize them at once, not by the use of arsenic, but with carbolic acid and cantharides. My method is to moisten a small pellet of cotton with carbolic acid and then apply to its undersurface a little powdered cantharides. One or two applications are usually sufficient to devitalize the pulp in a temporary tooth. When the pulp is found to be painless it is removed from the pulp-chamber, and as thoroughly as convenient from the canals ; a disk of thin platinum is then fitted over the opening leading from the cavity of decay in the pulp-chamber. To hold this disk in position a little gutta-percha or gum-damar is placed upon its under surface; the whole is then warmed in a spirit lamp and tacked in position in the cavity. The cavity of decay is then filled with amalgam or phosphate of zinc, as may be thought preferable. After this has been done a small tap-hole is made with a small drill upon the buccal surface at the free margin of the gums, to allow the egress of any gases which may arise from the non-removal of any portion of the pulp. While I would condemn as heartily as anyone present a similar treatment for permanent molars, I am convinced, after many years of such treatment, that it is the best known for temporary teeth. It prevents the probability of subsequent pain and the too frequent result of abscesses. You will all bear me witness that a devitalized pulp in a temporary tooth means a subsequent abscess if this precaution of drilling is not observed, and many nights of suffering are spared the little patient if this practice is followed.

There is another operation upon the temporary molars which is too frequently overlooked, but the importance of which cannot be over-estimated. I allude to the free cutting of the distal surface of the second molar upon the appearance of the first permanent or sixth-year molar. When this operation is neglected, the mesial surface of the permanent molar is usually the seat of caries. As soon as the sixth-year molar has taken its position in the arch alongside of the second temporary molar, I cut away largely with a disk from its distal face, thus preventing three or four years of contact, which at this period of life is so fatal in its results.

I had not intended speaking of the temporary teeth with reference to their influence upon the permanent ones. I have never been of the opinion that the premature loss of the temporary set exerted a marked effect upon the position of the permanent ones in the matter of irregularity; but there is one mistake which dentists so frequently make that I believe it worthy of our consideration for a moment. I refer to the early removal of the temporary cuspids to make room for the permanent laterals. In my judgment a greater mistake cannot be made; and yet it is one which almost daily occurs. What is the result? The first permanent bicuspid, which precedes the permanent cuspids by several years, takes its position by the side of the permanent lateral, and no room is left for the cuspids. The choice then to make room for the coming tooth lies between the expansion or enlargement of the jaw, or the removal of the first bicuspid. To accomplish the first is usually an undertaking of no small moment; to resort to the other not infrequently gives, as the result, a deformity which is apparent as it is unnecessary. Allow the cuspids to remain, and trust to the rapid enlargement of the arch between the eighth and twelfth year to make room for the laterals.

And now, a few remarks with reference to the treatment of the permanent teeth in early life. Perhaps a better rendering of my subject would have been, "The Treatment of Young Teeth," for I did not intend confining my remarks to the temporary set. It has long been my opinion that young or uncalcified teeth demand a different treatment from those which have become hard from age.

Let us begin with the incisors, which are often the seat of caries soon after their eruption. Whatever they are found to approximate each other closely it has been my habit to separate them often with wooden wedges, and polish the approximal surfaces with emery tape, and finally with linen tape and pumice or chalk. I have seen the benefit of such treatment in two of my own children, aged respectively fifteen and twelve. When the four incisors had taken their position in the arch I inferred from their close contact that the approximal surfaces would certainly be the seat of caries if precautions were not taken to avoid it; therefore I began early the above treatment, and at intervals of a few months for several years have separated them and rubbed them freely with emery strips. The result has been most satisfactory. In the case of one of them the centrals had become the seat of minute cavities. These were filled with phosphate of zinc, which was allowed to become very hard before moisture was admitted. These fillings have been in nearly, if not quite, two years, and not the slightest wasting has yet taken place.

The practice of filling these young teeth so generally with gold is one which cannot be too strongly condemned. I believe the induction of thermal changes through gold fillings has been one of the most prolific causes of devitilization of the pulp. Were the plastic materials more generally used prior to the fifteenth year, better results would be obtained. Amalgam of the better grades for masticating surfaces, phosphate of zinc, gutta-percha, and tin foil for approximal surfaces, commend themselves to men of experience and close observation. In my own practice I rarely insert gold fillings in the anterior teeth before the child has attained the fifteenth year, and if the cavities are deep-seated, I take the precaution to interpose some non-conductor between the floor of the cavity and the gold.

The best operations of gold often fail in these young teeth, and how much wiser is it to fill such uncalcified teeth with some material easy of insertion, allowing the fillings to remain until the teeth have hardened and the patient attained an age when it will more patiently endure the fatigue incident to prolonged sittings.—*Dental Cosmos.*

